# Traumastem Powder in Treatment of Non-Traumatic Anterior Epistaxis in Emergency Department; a Randomized Clinical Trial

**Published:** 2020-10-04

**Authors:** Mahboob Pouraghaei, Sina Shafiee, Fahimeh Mesrian, Haniyeh Ebrahimi Bakhtavar, Farzad Rahmani

**Affiliations:** 1 Emergency Medicine Research Team, Tabriz University of Medical Sciences, Tabriz, East Azerbaijan, Iran.

**Keywords:** Epistaxis, Emergency Service, Hospital, Treatment Outcome, Patient Satisfaction

## Abstract

**Introduction:**

Various studies are being conducted because of the value of finding an appropriate medication to control bleeding in patients with epistaxis faster and more conveniently. This study aimed to compare the effect of Traumastem powder with routine tampons in treatment of non-traumatic epistaxis.

**Methods:**

This randomized clinical trial enrolled patients with epistaxis presenting to the emergency departments of two hospitals affiliated to Tabriz University of Medical sciences. Patients were divided into two groups using randomization software (intervention group: 107 patients, control group: 96 patients). Primary outcome variables included bleeding control time and patient satisfaction. Secondary outcome variable was recurrence of bleeding within the first 24 hours after treatment. Visual assessment scoring system was used to assess patient satisfaction.

**Results:**

Epistaxis was controlled in less than 5 minutes in 85 (79.4%) patients in the intervention group and 85 (88.5%) patients in the control group (P=0.058). Patient satisfaction in the intervention group was higher than that of the control group (P<0.05). In the intervention group, 10 patients experienced recurrence of epistaxis within 24 hours of treatment, while 9 patients in the control group experienced recurrence (P= 0.591).

**Conclusion:**

Based on the findings, bleeding control time was similar in the two groups, but patient satisfaction was higher in Traumastem group. It is concluded that Traumastem can conveniently control anterior epistaxis, but it is not successful in cases with severe bleeding.

## Introduction

Epistaxis is a common medical problem, especially in older adults. The highest prevalence occurs in hospitalized patients under 10 and over 40 years of age (1, 2) . Most of the epistaxis cases are idiopathic, and no primary risk factor is observed in 80%-90% of cases. The known causes of epistaxis include trauma (most common), nasal neoplasms, iatrogenic causes, and systemic causes (such as blood clotting, high blood pressure, inflammatory conditions, infectious diseases), medications (such as anticoagulants, antiplatelet, nasal sprays), and congenital anomalies of the nasal septum (1).

Various hemostatic dressings are used both in prehospital and hospital settings to control bleeding (5). Furthermore, advanced procedures such as nasal endoscopy are successfully used to control epistaxis (6). Zahed R. concluded that efficacy of topical use of tranexamic acid was higher than routine nasal tampon (7). Ala A. reported no difference between Celox^©^ and routine tampon in bleeding control of idiopathic anterior epistaxis (8).

Traumastem powder is an absorbable sterile powder, made of calcium hydrogen salt of oxidized cellulose, which is mostly used in outpatient settings and surgical fields to stop capillary and venous bleeding. It is even suitable for stopping small superficial bleeding from lacerations. Some of the outstanding features of this product include: it significantly increases the speed of clinical healing, has antibacterial effects, is completely absorbable, does not need to be removed from the wound, is non-irritating and hypoallergenic, does not cause any immunological processes, produces complete homeostasis within 2 minutes, significantly reduces patient treatment costs, and has no side effects reported so far (3, 4).

Traumastem powder is a new treatment method, but no research has so far been conducted to compare its therapeutic effects with other topical medications available for the treatment of epistaxis. The aim of this study was to compare this treatment with routine nasal packing (shrinkage + tampon) in the treatment of epistaxis in patients presenting to the emergency department.

## Methods

*Study Design and Setting*

This randomized clinical trial was conducted on patients with epistaxis presenting to the emergency departments of Imam Reza and Sina Teaching Hospitals affiliated to Tabriz University of Medical Sciences**, **during Dec 2018-Jan 2020**. **This study was approved by the Ethics Committee of Tabriz University of Medical Sciences (IR.TBZMED.REC.1397.480 on 10.05.2018). This study also was registered in Iranian Registry of Clinical Trials (IRCT20130224012592N6) on 08.10.2018.

*Participants*

Inclusion criteria were: patients aged 18 to 65 years who presented to the emergency departments of the mentioned hospitals with anterior epistaxis. Patients with coronary heart disease (INR>1.5), patients with multiple trauma, nasal trauma, arterial hemorrhage, shock, and patients who did not wish to participate in the study were excluded.

***Intervention***

**Figure 1:**
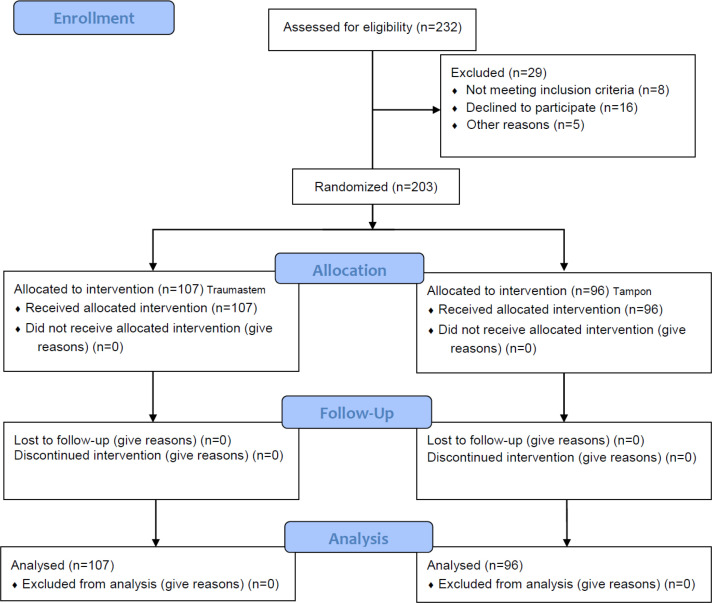
Flowchart of the study

To randomize samples, we used Random Allocation Software to allocate the patients into two groups of Traumastem and routine nasal packing. Intention to treat was our analysis strategy. Figure 1 shows the flowchart of the study.

After obtaining informed consent, patients' demographic information was recorded and initial measures were taken for the patient. At the same time, a blood sample was obtained to assess coagulation disorders (CBC-PT-PTT-INR). The Traumastem group received a puff of Traumastem (powder, 2 grams, BIOSTER, A.S Company, Veverská Bítýška, Czech Republic) on the bleeding site and then the patient was asked to press their nostrils. The tampon group underwent the conventional method to control bleeding. First, three band cottons soaked in 2% lidocaine with epinephrine(1:10000) were used to shrink the nostrils, then a band mesh stained with 3% tetracycline ointment was used for anterior nasal packing (9). If bleeding control failed in the Traumastem group, they received routine anterior nasal packing. 

***Measurements of outcomes***

Primary variables included bleeding control time and patient satisfaction. The secondary outcome variable was no recurrence of bleeding in the first 24 hours after treatment. Both groups of patients were assessed for bleeding control at 5-minute intervals, and bleeding control time was recorded. Patient satisfaction was assessed using Visual Analog Scale (VAS) in which score 1 meant dissatisfaction and score 10 presented the greatest satisfaction. Patient satisfaction was assessed on discharge from ED. The length of stay (LOS) of patients in the ED was collected. Recurrence of bleeding was assessed by calling the patients 24 hours after the treatment.

*Statistical analysis*

To calculate the sample size of the study, the success rate of anterior nasal tampon in anterior epistaxis control within 10 minutes was considered as 31%, based on Zahed’s study(7). Probability of controlling bleeding under 10 minutes using a Celox tampon cell for patients was considered 55% (∆ = 25%). By considering the power of 80% for the two-tailed test and using G power software (Version 3.1.9.4., Franz Faul, Universität Kiel, Germany), the number of samples required for each group was calculated to be 93.

Data were analyzed using SPSS software (version 22.0, Chicago, USA). Descriptive statistics including quantity and percentage were used for qualitative variables, and mean and standard deviation were used for quantitative variables. The normal distribution of quantitative variables was analyzed using Kolmogorov-Smirnov test. The p value of this test was <0.05. Thus, we used Mann-Whitney U test to analyze the quantitative variables. Chi-square test was used to analyze qualitative variables. Linear regression was used to analyze the relationship between quantitative variables. A p-value less than 0.05 was considered significant.

## Results

In this study, 203 patients (107 patients in the Traumastem group, 96 patients in the tampon group) were included. Mean ± SD of patients’ age was 51.90±16.48 years (56.2% male). Table 1 shows the comparison of demographics, vital signs, and laboratory variables of patients between the two groups.

Table 2 demonstrates the comparison of the data related to bleeding control and patient satisfaction in the two groups. As shown in Table 2, there is no significant statistical difference between the two groups regarding bleeding control time (P value=0.764), but patient satisfaction was higher in Traumastem group, and recurrence of bleeding within 24 hours of treatment was not significantly different between the 2 groups (P= 0.591).

**Figure 2:**
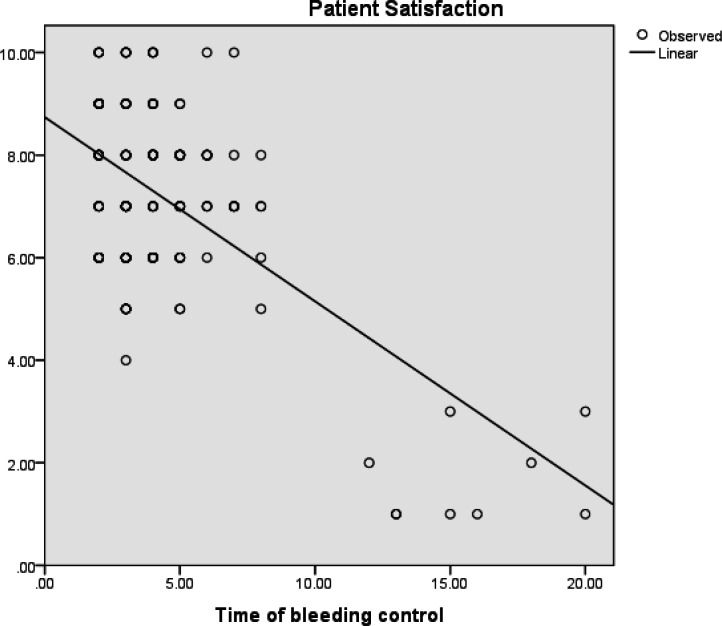
Relationship between patient satisfaction level (Vertical axis) and time of bleeding control (Horizontal axis)

To evaluate the relationship between patient satisfaction level and time of bleeding control, we used linear regression (Figure 2). Based on the results of this test, there was a significant statistical correlation between these two variables, as satisfaction level was higher when time of bleeding control was shorter (R=0.356, P value<0.001).

In 5 cases in the early moments of Traumastem use, this agent led to sneezing, so we tried to use a routine nasal tampon to stop bleeding, and these patients were excluded from the study.

To calculate the absolute risk reduction (ARR), relative risk (RR), and the number need to harm (NNH) of the new treatment of epistaxis, we used the number of patients with successful bleeding control under 5 minutes in each group. The results showed that the ARR, RR, and NNH were -0.09 (95% CI -0.19 - 0.074), 1.79 (95% CI 0.92 - 3.5) and 10.99 (95% CI -5.24 - 116.37), respectively.

**Table 1:** Comparison of demographic data, vital signs, and laboratory variables of patients between the two groups

**Variables**	**Traumastem n = 107**	**Tampon n = 96 **	**P **
Age (years)	60.00 (45.00-62.00)	54.5 (36.00-62.75)	0.076^*^
**Gender **			
Male	63 (58.9%)	51 (53.1%)	0.247^&^
Female	44 (41.1%)	45 (46.9%)	
**Epistaxis history **			
Yes	32	33	0.298^&^
No	75	63	
**Vital signs**			
MAP^#^ (mmHg)	90 (87-100)	90 (90-100)	0.301^*^
Heart rate (/minute)	81 (72-93)	85 (75-90)	0.376^*^
**Laboratory data**			
Platelet (/mm^3^)	230000 (190000-270000)	215000 (167250-298000)	0.494^*^
PT^#^(second)	13 (13-13)	13 (13-13)	0.410^*^
PTT^#^ (second)	33 (32-35)	33 (32-35)	0.287^*^
INR^#^	1 (1-1.10)	1 (1-1.10)	0.452^*^

#MAP: Mean Arterial Pressure; PT: Prothrombin Time; PTT: Partial Thromboplastin Time; INR: International Normalized Ratio.

Data are presented as median (IQR 25-75%) or number (%). *: Mann U Whitney; ^&^: Chi square.

**Table 2:** Comparison of data on bleeding control and patients’ satisfaction in the two groups

**Variables**	**Traumastem n= 107**	**Tampon n=96**	**P **
**Bleeding control time (minute)**			
Median (IQR 25-75%)	4.00 (3.00-5.00)	4.00 (3.00-4.00)	0.764^*^
**Satisfaction level**			
Median (IQR 25-75%)	8.00(8.00-9.00)	6.00 (6.00-7.00)	<0.001^*^
**Length of stay in ED (minute)**			
Median (IQR 25-75%)	85.00 (45.00-110.00)	60.00 (45.00-88.00)	0.095^*^
**Control of bleeding** **≤** **5minutes**			
Yes	85 (86.7)	85 (88.5)	0.058^&^
No	22 (20.6)	11 (11.5)	
**Re-bleeding after 24 hours**			
Yes	10 (9.3)	9 (9.4)	0.591^&^
No	97 (90.7)	87 (90.6)	

ED: emergency department; *: Mann U Whitney; ^&^: Chi square.

## Discussion

In this study, we evaluated the efficacy of Traumastem powder for bleeding control in epistaxis. There was no significant statistical difference between the two groups regarding bleeding control time (p value: 0.764), but there was significant difference in patients’ satisfaction rate (p value˂0.001).

Epistaxis, which is most often benign, is one of the common complaints in emergency departments (10). Most of the epistaxis cases are treated with simple measures at home or out of the hospital, but some of them require hospital admission and professional medical treatments (11). Many treatment modalities have been introduced to control bleeding in epistaxis (12). Some of the anterior nasal bleeding treatments include direct nasal pressure, chemical cauterization, thrombogenic foams and gels, and anterior and posterior nasal packing (8, 9, 13, 14). Different studies have also been conducted to evaluate new treatments and compare their efficacy and patient satisfaction (6-8).

Recently, oxidized cellulose-containing hemostatic powders have been introduced to control capillary, venous, and small arterial hemorrhages in inaccessible areas. Different studies have evaluated the efficacy of these products (15-19).

Zou Y. compared endoscopic surgery and nasal packing in posterior epistaxis, and reported no significant differences between the two methods in the basic features, but the degree of adhesion of the nasal cavity was significantly lower in the surgery group compared to the nasal packing group (6). Farnetti P. compared topical sclerotherapy using Lauromacrogol with nasal packing in treatment of epistaxis, and reported the recurrence rate to be significantly higher in the nasal packing group compared to the other group (1). Murray S. et al. concluded that Floseal was a comfortable, effective, and cost-effective alternative in treatment of resistant epistaxis compared to the old packing method in patients with normal coagulation profile (2). Zahed R. concluded that tranexamic acid had a better effect on epistaxis control compared to other methods (7). Ala A. et al. reported no better results with Celox^©^ bands in terms of bleeding control time in comparison to routine tampon, but satisfaction level was higher in the Celox^©^ group (8).

In this study, 107 patients were included in the Traumastem group (intervention) and 96 patients were allocated to the tampon group (control). There were no significant statistical differences between the two groups regarding demographics, primary vital signs and laboratory data (P value˃0.05). In the acute treatment of epistaxis and rebleeding rate within 24 hours of treatment, Traumastem was not better than routine anterior nasal packing.

## Limitations

The significant limitation of our study was in the blinding stage, considering the differences between the types of methods used, it was impossible to blind the patients or physicians to the methods. In some of the patients with severe bleeding, it took Traumastem a long time to control bleeding, which negatively affected these patients’ satisfaction. In some patients, in the early moments of using the powder, it induced sneezing, which was a limitation for its use.

## Conclusion

Based on the findings, bleeding control time was similar in the two groups, but patient satisfaction was higher in the Traumastem group. In addition, recurrence rate of bleeding within 24 hours of treatment was similar in both groups. We need further studies on the efficacy and applicability of (Traumastem) in routine practice to assess the generalizability (external validity, applicability) of the results of the present study. 

## References

[1] Farneti P, Pasquini E, Sciarretta V, Macrì G, Gramellini G, Pirodda A (2016). Comparison of Local Sclerotherapy With Lauromacrogol Versus Nasal Packing in the Treatment of Anterior Epistaxis. Clin Exp Otorhinolaryngol.

[2] Murray S, Mendez A, Hopkins A, El-Hakim H, Jeffery CC, Côté DWJ (2018). Management of Persistent Epistaxis Using Floseal Hemostatic Matrix vs traditional nasal packing: a prospective randomized control trial. J Otolaryngol Head Neck Surg.

[3] Czech Republic Bioster a.s.

[4] Zboril P, Vyslouzil K, Klementa I, Starý L, Skalický P, Růzicka V (2010). Ambulantní excize perianálnich duplikatur [Ambulatory excision of perianal duplicatures]. Rozhl Chir.

[5] Granville-Chapman J, Jacobs N, Midwinter MJ (2011). Pre-hospital haemostatic dressings: a systematic review. Injury.

[6] Zou Y, Deng YQ, Xiao CW, Kong YG, Xu Y, Tao ZZ (2015). Comparison of outcomes between endoscopic surgery and conventional nasal packing for epistaxis in the posterior fornix of the inferior nasal meatus. Pakistan journal of medical sciences.

[7] Zahed R, Moharamzadeh P, Alizadeharasi S, Ghasemi A, Saeedi M (2013). A new and rapid method for epistaxis treatment using injectable form of tranexamic acid topically: a randomized controlled trial. The American journal of emergency medicine.

[8] Ala A, Rahmani F, Shirzadegan S, Ebrahimi Bakhtavar H, Mehdizadeh Esfanjani R (2018). Non - traumatic Epistaxis Management Using Celox® Dressing: A Randomized Clinical Trial. Iran Red Crescent Med J.

[9] McGinnis HD, Tintinalli JE (2020). Nose and Sinuses. Tintinalli’s Emergency Medicine.

[10] Sarhan NA, Algamal AM (2015). Relationship between epistaxis and hypertension: A cause and effect or coincidence?. J Saudi Heart Assoc.

[11] Alyahya K, Alsaad S, Alsuliman S, Alsuliman N (2019). Awareness about first aid management of epistaxis among medical students in Kingdom of Saudi Arabia. J Family Med Prim Care.

[12] Newton E, Lasso A, Petrcich W, Kilty SJ (2016). An outcomes analysis of anterior epistaxis management in the emergency department. J Otolaryngol Head Neck Surg.

[13] Bachelet JT, Bourlet J, Gleizal A (2013). Hemostatic absorbable gel matrix for severe post-traumatic epistaxis. Rev stomatol Chir Maxillofac Chir Orale.

[14] Toner JG, Walby AP (1990). Comparison of electro and chemical cautery in the treatment of anterior epistaxis. J Laryngol Otol.

[15] Hutchinson RW, Werrlein S, Johns DB, Zhang G, Clymer JW, Kocharian R (2015). An In Vivo Comparison of Hemostatic Gelatin Matrix Products in a Porcine Spleen Biopsy-punch Model. Surgical technology international.

[16] MacDonald MH, Wang AY, Clymer JW, Hutchinson RW, Kocharian R (2017). An in vivo comparison of the efficacy of hemostatic powders, using two porcine bleeding models. Medical devices (Auckland, NZ).

[17] Martyn D, Meckley LM, Miyasato G, Lim S, Riebman JB, Kocharian R (2015). Variation in hospital resource use and cost among surgical procedures using topical absorbable hemostats. Clinicoecon Outcomes Res.

[18] Rembe J-D, Böhm JK, Fromm-Dornieden C, Schäfer N, Maegele M, Fröhlich M (2015). Comparison of hemostatic dressings for superficial wounds using a new spectrophotometric coagulation assay. J Transl Med.

[19] Wright JD, Ananth CV, Lewin SN, Burke WM, Siddiq Z, Neugut AI (2014). Patterns of use of hemostatic agents in patients undergoing major surgery. J Surg Res.

